# Comparative Secretory Efficiency of Two Chitosanase Signal Peptides from *Bacillus subtilis* in *Escherichia coli*

**DOI:** 10.1007/s12275-024-00186-1

**Published:** 2024-11-25

**Authors:** Tae-Yang Eom, Yehui Gang, Youngdeuk Lee, Yoon-Hyeok Kang, Eunyoung Jo, Svini Dileepa Marasinghe, Heung Sik Park, Gun-Hoo Park, Chulhong Oh

**Affiliations:** 1 Jeju Bio Research Center, Korea Institute of Ocean Science and Technology, Jeju, 62632 Republic of Korea; 2 Department of Marine Technology and Convergence Engineering, University of Science and Technology, Daejeon, 34113 Republic of Korea

**Keywords:** Signal peptide, Chitosanase, Recombinant protein, Expression, Secretion, Sec pathway, *E. coli*, *Bacillus subtilis*, Cu,Zn-Superoxide dismutase

## Abstract

The production of recombinant proteins in *Escherichia coli* is often challenged by cytoplasmic expression due to proteolytic degradation and inclusion body formation. Extracellular expression can overcome these problems by simplifying downstream processing and improving protein yields. This study aims to compare the efficiency of two *Bacillus subtilis* chitosanase signal peptides in mediating extracellular secretion in *E. coli*. We identified a naturally occurring mutant signal peptide (mCsn2-SP) from *B. subtilis* CH2 chitosanase (CH2CSN), which is characterized by a deletion of six amino acids in the N-region relative to the signal peptide (Csn1-SP) from *B. subtilis* CH1 chitosanase (CH1CSN). The CH1CSN and CH2CSN genes were cloned into the pET-11a vector and protein secretion was evaluated in *E. coli* BL21(DE3) host cells. Expression was induced with 0.1 mM and 1 mM isopropyl β-d-1-thiogalactopyranoside (IPTG) at 30 °C for one and three days. CH2CSN showed higher secretion levels compared to CH1CSN under all experimental conditions, especially with 0.1 mM IPTG induction for 3 days, which resulted in a 2.37-fold increase in secretion. Furthermore, it was demonstrated that mCsn2-SP is capable of secreting human Cu,Zn-superoxide dismutase (hSOD) in *E. coli* BL21(DE3) and successfully translocating it to the periplasmic region. This study represents the inaugural investigation into the utilisation of a naturally modified signal peptide, thereby corroborating the assertion that signal peptide deletion variants can influence protein secretion efficiency. Furthermore, the findings substantiate the proposition that such variants can serve as a viable alternative for the secretion of heterologous proteins in *E. coli*.

**Supplementary Information:**

The online version contains supplementary material available at 10.1007/s12275-024-00186-1.

## Introduction

The industrial production of recombinant proteins secreted by *E. coli* is essential in the biotechnology industry (Yoon et al., 2010). *E. coli* has advantageous properties, such as rapid growth to high cell densities on a low-cost carbon source, and serves as a platform for recombinant protein production (Baneyx & Mujacic, 2004). Due to its extensive use as a model system, *E. coli* genetics have been extensively characterized, resulting in the development of numerous tools that aid in chromosome engineering, gene replication, and gene expression (Mergulhao et al., 2005). Recombinant proteins can be targeted to different compartments within *E. coli*, including the cytoplasm, periplasm, and extracellular space. However, cytoplasmic expression of recombinant proteins is challenging. Proteins expressed in the cytoplasm may be susceptible to degradation by cellular proteases or accumulate as insoluble inclusion bodies, rendering them inactive (Mergulhao & Monteiro, 2007). In addition, cytoplasmic expression of certain proteins can have toxic effects on host cells, leading to reduced protein yields or even cell death (Mierendorf et al., 1998). In contrast, extracellular expression in *E. coli* offers several advantages for the production of recombinant proteins, including simplified downstream processing, reduced host cell contamination, high expression levels, reduced protein degradation, and increased solubility (Choi & Lee, 2004). *E. coli* has two major secretory pathways, the Sec and Tat pathways (Costa et al., 2015). The Sec pathway, which facilitates the transport of proteins from the cytoplasmic membrane, was the first secretory pathway identified in bacteria (Beckwith, 2013). This pathway has demonstrated the ability to translocate proteins in an unfolded state, thereby facilitating the efficient export of target proteins (Crane & Randall, 2017). In contrast, the Tat pathway has the distinct ability to transport fully folded proteins across the cytoplasmic membrane (Choi & Lee, 2004).

Chitosanase (EC 3.2.1.132) catalyzes the hydrolysis of β-1,4-glucosidic linkages in the polymeric chitosan, a type of polysaccharide. Chitosan is a high molecular weight polymer that requires enzymatic degradation for human consumption. It is broken down into chitosan oligosaccharides by extracellular chitosanases found in various bacteria. Chitosan oligosaccharides offer more significant biological functions compared to chitosan, including higher levels of antibacterial, antioxidant, blood cholesterol reduction, blood pressure improvement, infection prevention, arthritis control, and antitumor effects (Yabuki et al., 1988). The GH-46 type chitosanases, especially those derived from Bacillus and Streptomyces, have been extensively studied for their catalytic functions, enzymatic mechanisms, and protein structures (Wang et al., 2008).

Previously, our studies have shown that chitosanase derived from *B. subtilis* CH1 and *B. subtilis* CH2 are secreted into the extracellular space of *B. subtilis* (Oh & Lee, 2006; Oh et al., 2011). In this study, we compared the efficiency of two chitosanase signal peptides from *B. subtilis* in mediating the extracellular secretion of chitosanase when introduced into *E. coli*.

## Materials and Methods

### Molecular Characterization of Chitosanases from *B. subtilis* Strains

Signal peptides were analyzed using SignalP version 6.0, available at the Health Tech website (https://services.healthtech.dtu.dk/services/SignalP-6.0/) (Teufel et al., 2022). The N-region net charge of signal peptides predicted using peptide calculator (https://www.pepcalc.com/). Conserved domains and active sites within the protein sequences were identified using the protein BLAST tool from the National Center for Biotechnology Information (NCBI) (https://blast.ncbi.nlm.nih.gov/Blast.cgi?PROGRAM=blastp&PAGE_TYPE=BlastSearch&BLAST_SPEC=&LINK_LOC=blasttab&LAST_PAGE=blastp). Molecular weights were predicted using the Compute pI/Mw tool available at ExPASy (https://web.expasy.org/compute_pi/). The nucleotide and amino acid sequences of chitosanase from *B. subtilis* strain CH2 were compared with those of strains 168 and CH1 using Clustal multiple sequence alignments in the DNADynamo program (BLUE TRACTOR SOFTWARE Ltd, North Wales, UK).

### Genomic DNA Extraction

*B. subtilis* strains CH1 and CH2 were obtained from Jeju National University (Jeju, Korea). These strains were cultured in 4 mL of Luria–Bertani (LB) broth (BD, USA) at 30 °C for 16 h. After incubation, cells were harvested by centrifugation. The cell pellets were then resuspended in 200 μL lysozyme (Takara Bio Inc., Japan) solution (20 mg/mL in 20 mM Tris–Cl, pH 8.0) and incubated at 37 °C for 30 min. To the lysed cells, 100 μg/mL proteinase K (Bioneer Co., Korea) and 30 μL 10% sodium dodecyl sulfate (Merck, Germany) were added. The mixture was further incubated at 56 °C for 30 min. For DNA extraction, an equal volume of phenol/chloroform/isoamyl alcohol (25:24:1) (Bioneer Co., Korea) was added to the lysate, followed by centrifugation at 13,000×*g* at 4 °C for 2 min. The aqueous phase was carefully transferred to a new tube to which 3 M Na-acetate (1/10 volume) was added. Two times the volume of 100% ethanol was added, the mixture was inverted several times and stored at − 20 °C for 2 h to precipitate the DNA. The samples were then centrifuged at 13,000×*g* for 30 min at 4 °C, and the supernatant was discarded. The DNA pellets were washed with 70% ethanol and centrifuged again at 13,000×*g* at 4 °C for 10 min. After discarding the supernatant, the pellets were dried at 20 °C for 30 min. Finally, the dried genomic DNA was resuspended in 100 μL distilled water.

### Gene Cloning of Chitosanases from *B. subtilis* CH1 and CH2

Primers were designed to amplify the coding region, including the signal sequences, from the genomic DNA of *B. subtilis* strains CH1 and CH2. The forward primer, CH-F (5′-GAGA**CATATG**AAAATCAGTATGCAAAAAGC-3′), contained a *Nde*I site, and the reverse primer, CH-R (5′-GAGA**GGATCC**TTATTTGATTACAAAATTACCGTA-3′), contained a *Bam*HI site. The PCR mixture consisted of 5 μL of 10X Ex *Taq* DNA polymerase buffer, 8 μL of 2.5 mM dNTP mixture, 5 μL of 25 mM MgCl2, 100 pmol of each primer, 1 μg of genomic DNA, and 3 units of Ex *Taq* DNA polymerase (Takara Bio Inc., Japan) in a final volume of 50 μL. PCR cycling conditions were set as follows: initial denaturation at 94 °C for 5 min, followed by 30 cycles of 94 °C for 1 min, 55 °C for 30 s, and 72 °C for 1 min, with a final extension at 72 °C for 5 min. PCR products were verified by 1% agarose gel electrophoresis and purified using a gel purification kit (Bioneer Co., Korea). The verified PCR products and the pET-11a vector (Novagen, USA) were digested with *Nde*I and *Bam*HI restriction enzymes (Takara Bio Inc., Japan) according to the manufacturer's instructions. The digested fragments were then ligated into the vector using T4 DNA ligase (Takara Bio Inc., Japan). The ligation mixture was transformed into *E. coli* DH5alpha cells by the heat shock method. Transformed colonies were selected on LB agar plates containing 100 μg/mL ampicillin (Merck, Germany) and incubated in 4 mL LB broth at 37 °C overnight with shaking at 180 rpm. Plasmid DNA (pET11a-CH1 and pET11a-CH2) was extracted from the cultured cells using an Accuprep Nano-plus Plasmid Mini Extraction Kit (Bioneer Co., Daejeon, South Korea). The nucleotide sequences of the plasmids were confirmed by sequencing at Macrogen (Korea).

### Overexpression of Chitosanases from *B. subtilis* CH1 and CH2 in *E. coli*

The recombinant plasmids pET11a-CH1 and pET11a-CH2 were transformed into *E. coli* BL21(DE3) (Novagen, USA) cells. Transformed cells were plated on LB agar (BD, USA) containing 100 μg/mL ampicillin and incubated at 37 °C for 16 h. Single colonies were inoculated into 20 mL LB broth supplemented with 100 μg/mL ampicillin and cultured at 37 °C with shaking at 180 rpm. When the optical density at 600 nm reached 0.4, recombinant protein expression was induced by the addition of IPTG (Merck, Germany) at concentrations of 0.1 mM and 1 mM. Cultures were then incubated at 30 °C with shaking at 180 rpm for three days. Samples were collected on day 1 and day 3 post-induction, with 1 mL aliquots taken for each time point. Samples were centrifuged at 7000×*g* for 5 min to separate the pellet and supernatant. The collected pellets were resuspended in 1 mL cold distilled water and disrupted using a sonicator (Qsonica, USA) to release intracellular contents. The cell lysates and supernatants were analyzed by 10% SDS–polyacrylamide gel electrophoresis (PAGE) using MOPS buffer (Thermo Fisher Scientific, USA). The gels were then stained with Coomassie Brilliant Blue (Merck, Germany) to visualize proteins.

### Protein Measurement

Protein quantification was performed using ImageJ software (NIH, USA). Bovine serum albumin (BSA) protein (Thermo Fisher Scientific, USA) was used as the standard for protein quantification.

### Chitosanase Assay

Chitosanase activity was measured using soluble chitosan as the substrate. To prepare soluble chitosan, 1 mL of acetic acid was added to 80 mL of distilled water, and 1 g of chitosan (Merck, Germany) was dissolved in this mixture. The pH of the solution was then adjusted to 5.5 using 10 N NaOH. Subsequently, the volume was augmented to 100 mL through the addition of distilled water. Subsequently, the enzyme solution was combined with 1% soluble chitosan, thereby initiating the reaction. The reaction was conducted at 50 °C for 10 min, in accordance with the methodology described by Rondle and Morgan (1955). One unit (U) of enzyme activity was defined as the amount of enzyme required to produce 1 μmol of reducing sugar per minute. Glucosamine was employed as the standard for quantification.

### Application of mCsn2-SP to hSOD

The hSOD nucleotide sequence linked with mCsn2-SP was genetically modified through the implementation of codon optimization using the codon optimization tool (https://sg.idtdna.com/CodonOpt), which was submitted to GenBank under the accession numbers OR661283. The gene sequence was synthesized and cloned into the pTOP Blunt V2 vector by Macrogen (Korea). Primers were designed for the purpose of amplifying the synthesized gene and inserting it into the pET-11a vector via the In-Fusion method. The forward primer was designed as 5′-TAGCATAATATA**CATATG**AAAATTTCTATGCAG-3′, while the reverse primer was designed as 5′-TTGTTAGCAGCC**GGATCC**TTACTGTGCGATCC-3′. The polymerase chain reaction (PCR) mixture consisted of 5 μL of 10× Ex *Taq* DNA polymerase buffer, 8 μL of a 2.5 mM dNTP mixture, 20 pmol of each primer, 10 ng of template, and 3 units of Ex *Taq* DNA polymerase, resulting in a final volume of 50 μL. The PCR cycling conditions were set as follows: an initial denaturation at 94 °C for 5 min was performed, followed by 2 cycles of 94 °C for 30 s, 50 °C for 30 s, and 72 °C for 40 s. Subsequently, 15 cycles of 94 °C for 30 s, 60 °C for 30 s, and 72 °C for 40 s were conducted, followed by a final extension at 72 °C for 5 min. The PCR product was purified using a gel purification kit. The PCR product and the pET-11a vector digested with *Nde*I and *Bam*HI were fused using the EZ-Fusion™ Cloning Kit (Enzynomics, Korea) according to manual. It was then subcloned into *E. coli* DH5α as described above and finally transformed into *E. coli* BL21(DE3) cells. The cells were cultured in 100 mL of LB broth containing 100 μg/mL ampicillin at 37 °C with shaking at 180 rpm. At an optical density of 0.7 at 600 nm, protein expression was induced by the addition of IPTG to a final concentration of 1 mM. The induction was conducted at 20 °C with agitation at 180 rpm for 24 h. The inducted sample 1 mL was centrifuged at 7000×*g* for 5 min to separate the pellet and supernatant. The pellets were resuspended in 1 mL of cold distilled water and disrupted using a sonicator. The lysed cells were subjected to centrifugation at 15,000×*g* for 1 h with the objective of separating the soluble and insoluble proteins. The supernatant was then transferred to a new tube, while the remaining pellet was diluted with 1 ml of distilled water. Additionally, periplasmic protein isolation was conducted using 50 ml of induced cells, in accordance with the established protocol for the osmotic shock method (French et al., 1996). The proteins were analyzed by 10% SDS- PAGE using MES buffer (Thermo Fisher Scientific, USA).

## Statistical Analysis

All experiments were performed in triplicate, and the data were analyzed using GraphPad Prism version 8 (GraphPad Software, USA). Results are presented as the mean ± standard deviation. Significant differences between the groups were determined using analysis of variance (ANOVA), with a p value of less than 0.05 considered statistically significant.

## Results

### Molecular Characterization of Chitosanase

**Fig. 1:**
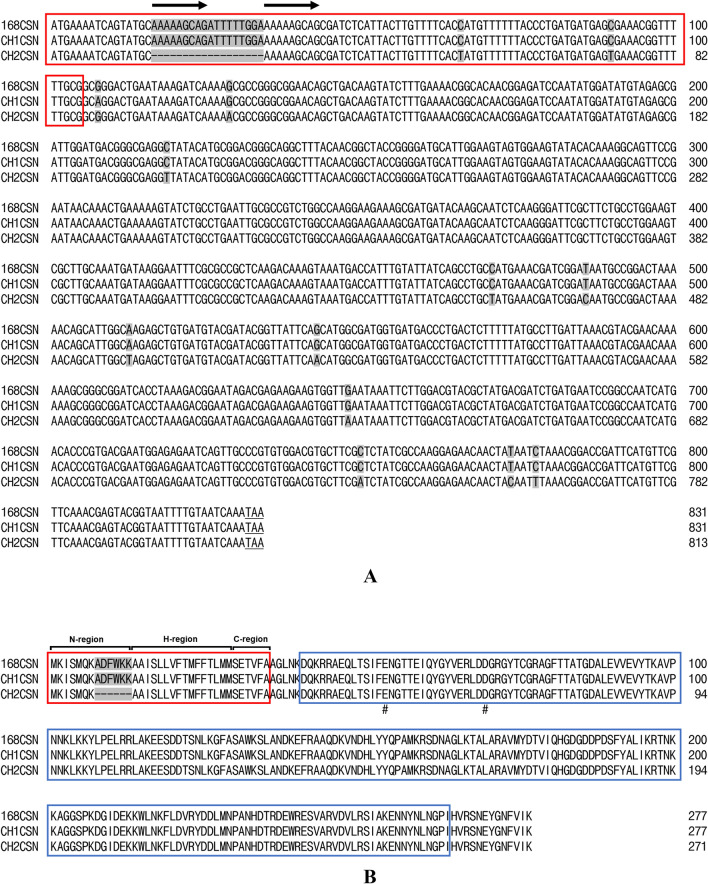
The nucleotide and amino acid sequences analysis of CH2CSN compared to 168CSN and CH1CSN. **A** Nucleotide sequences analysis. Red box: signal sequence, highlight in gray: sequence difference, arrow: repeat sequence; **B** Amino acid sequences analysis. Red box: signal peptide, highlight in gray: sequence difference, blue box: GH46 chitosanase domain; #: catalytic residues (active sites)

The nucleotide (nt) sequence of the CH1CSN gene contains an 831-base pair (bp) open reading frame (ORF) encoding a chitosanase protein of 277 amino acid (aa) residues (GenBank accession number GU001718). Conversely, the nt sequence of the CH2CSN gene has an 813-bp ORF encoding a chitosanase protein of 271 aa residues (GenBank accession number GU001716). Figure 1 show the nt and aa sequences in comparison with the chitosanase from *B. subtilis* strain 168 (168CSN). The signal peptides of CH1CSN and CH2CSN, predicted to be located in the N-terminal region, consist of 35 aa and 29 aa, respectively, and were identified as Sec pathway signal peptides using the SignalP program (Fig. S1). Despite an 11 bp difference in the mature chitosanase N-terminal sequences of CH1CSN and CH2CSN, their amino acid sequences remained identical. Notwithstanding, the signal sequence exhibited 20 bp mismatches in the nt sequence, 18 nt sequences were deleted, resulting in the removal of six amino acids, and two nt sequences were replaced, though this did not affect the amino acids. Of interest, a repeated sequence (AAAAAGCAG) was identified in the signal sequences of 168CSN and CH1CSN, and based on these sequences, we were able to confirm that some sequences were deleted in the signal sequence of CH2CSN (Fig. 1). In the presence of the signal peptide, the predicted molecular weight of CH1CSN is 31.5 kDa, whereas in its absence, it is 27.4 kDa. Similarly, the predicted molecular weight for CH2CSN is 30.7 kDa with the signal peptide and 27.4 kDa without it. All chitosanase sequences lacked cysteine residues and predicted the GH46 domain including two catalytic residues using the protein BLAST tool (Fig. 1B).

### Expression and Extracellular Secretion Analysis of Chitosanase in *E. coli*

**Fig. 2:**
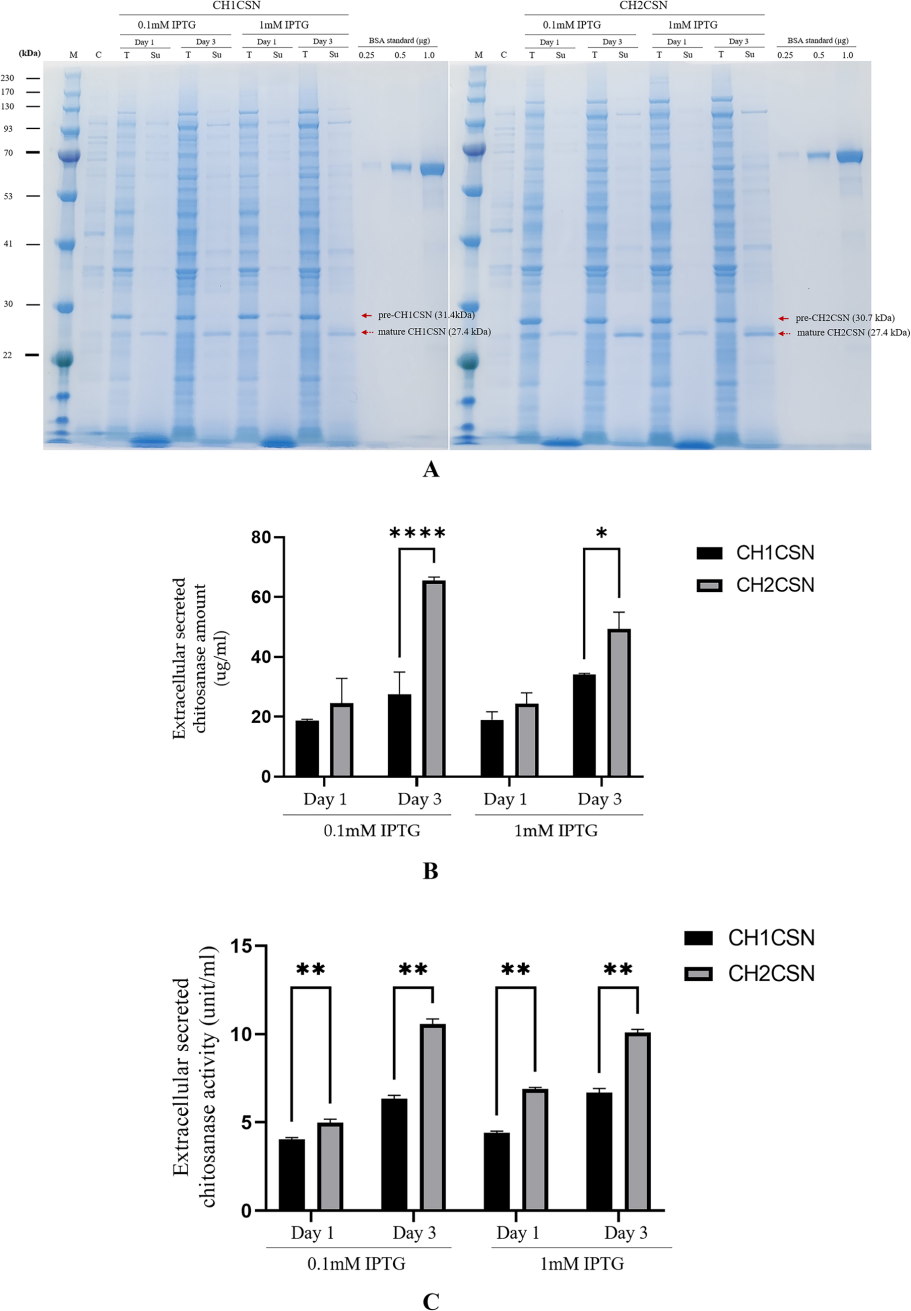
A comparative study of recombinant chitosanase expression using Csn1-SP and mCsn2-SP in response to different IPTG concentrations and times. **A** The analysis of protein expression patterns using SDS-PAGE. M: molecular mass standard marker, C: control (only pET11a vector induction), T: total cellular protein, Su: supernatant, BSA standard: bovine serum albumin protein for protein quantification; **B** Comparison of extracellular secreted chitosanase levels between CH1CSN and CH2CSN; **C** Comparison of secreted CH1CSN and CH2CSN activities. Asterisks indicate a significant mean difference between the test groups (mean ± standard deviation, n = 3; analysis of variance, p < 0.05)

To express CH1CSN and CH2CSN from *B. subtilis* in *E. coli* and evaluate their secretion efficiency, the chitosanase genes including the signal sequences were amplified by PCR and cloned into the pET11a vector. The constructs were then transformed into *E. coli* BL21(DE3) cells. The chitosanase proteins were induced by different IPTG concentrations (0.1 and 1 mM) and different times (1 and 3 days) at 30 °C. SDS-PAGE was employed to differentiate chitosanase proteins based on the cleavage status of their signal peptides. Proteins with intact signal peptides remained in the cytoplasm, while those with cleaved signal peptides were located in the periplasm and secreted extracellularly (Fig. 2A). Quantitative analysis of these proteins was conducted using image analysis software, and the detailed results are presented in Table 1. The data confirm that the signal peptides not only facilitated the export of chitosanase from the cytoplasm to the periplasm but also enabled its subsequent secretion outside the cell. The induction of CH1CSN expression with 0.1 mM and 1 mM IPTG resulted in an increase in protein secretion on day 3 compared to day 1. In the case of CH2CSN, secretion levels were found to be comparable at both IPTG concentrations on day 1. However, on day 3, a higher level was observed at 0.1 mM than at 1 mM IPTG (Fig. 2). It is noteworthy that the concentration of chitosanase was significantly elevated in the CH2CSN group relative to the CH1CSN group on day 3 with 0.1 mM IPTG. Similarly, chitosanase secretion levels were elevated in the CH2CSN group on day 3 with 1 mM IPTG (Fig. 2, Table 1). The total expression of chitosanase, which includes cytoplasmic, and extracellular components, was highest at 117.28 μg/mL when CH2CSN was treated with 0.1 mM IPTG for 3 days to induce expression. These results confirmed that a naturally mutated signal peptide from *B. subtilis* affected the efficiency of recombinant protein secretion in *E. coli*. The activity of CH1CSN was observed to be 4.04 U/mL and 6.35 U/mL on day 1 and day 3, respectively, at a 0.1 mM IPTG concentration. At a 1 mM IPTG concentration, the activity was observed to be 4.41 U/mL on day 1 and 6.69 U/mL on day 3. The activity of CH2CSN at a 0.1 mM IPTG concentration was recorded as 4.99 U/mL and 10.56 U/mL on day 1 and day 3, respectively. At a 1 mM IPTG concentration, the activity was 6.88 U/mL on day 1 and 10.09 U/mL on day 3 (Fig. 2C).

**Table 1:** Quantitative measurement of chitosanase secretion from CH1CSN and CH2CSN (mean ± standard deviation, n = 3)

	0.1 mM IPTG		1 mM IPTG	
	day 1	day 3	day 1	day 3
CH1CSN (μg/ml)				
Cytoplasm	48.04 ± 0.87	38.80 ± 2.87	47.08 ± 1.45	33.51 ± 0.51
Supernatant	18.75 ± 0.44	27.60 ± 7.39	18.95 ± 2.77	34.12 ± 0.38
CH2CSN (μg/ml)				
Cytoplasm	60.70 ± 1.92	51.80 ± 1.93	41.08 ± 1.90	28.49 ± 2.82
Supernatant	24.52 ± 8.29	65.48 ± 1.22	24.37 ± 3.65	49.31 ± 5.65

### Expression of mCsn2-SP Linked hSOD in *E. coli*

**Fig. 3:**
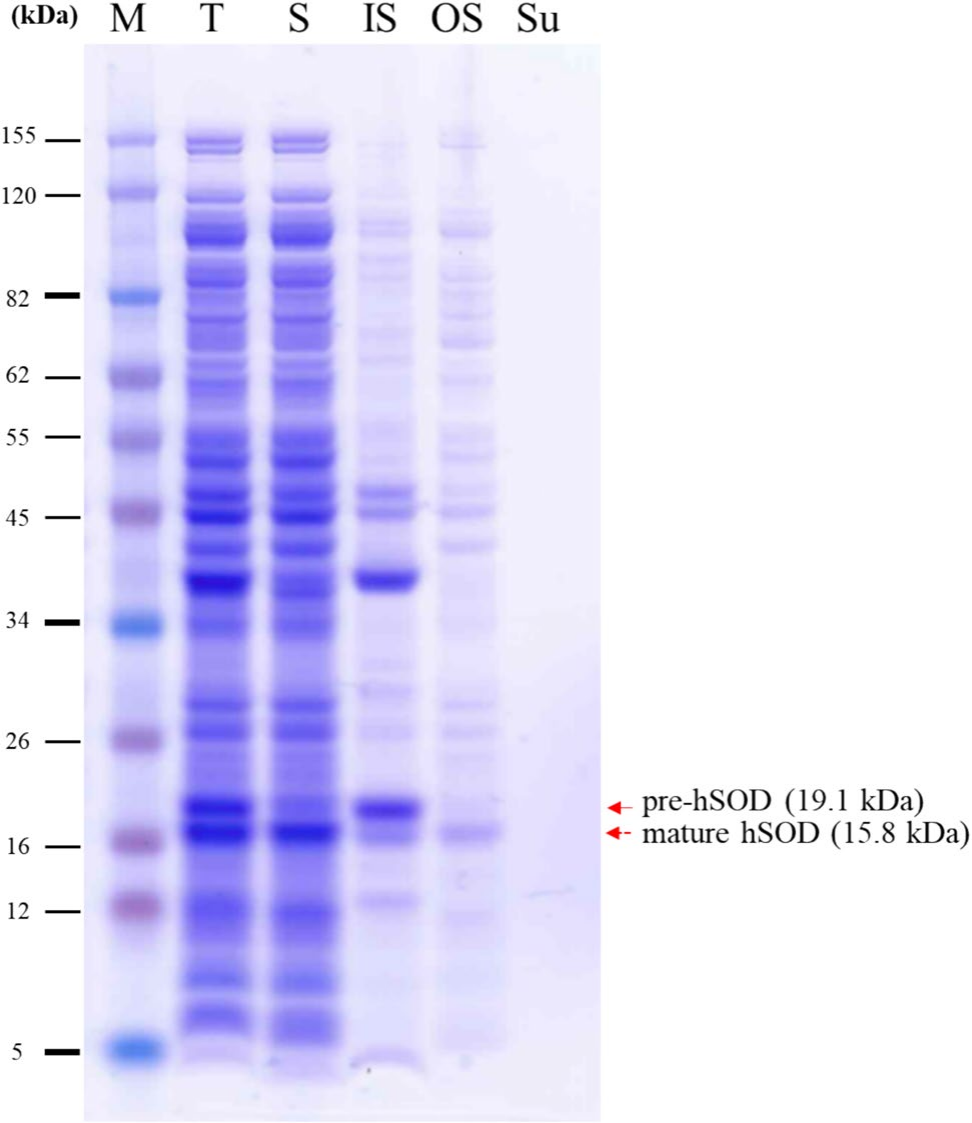
Recombinant hSOD expression analysis using mCsn2-SP. *M* protein marker, *T* total cellular protein, *S* soluble protein, *IS* insoluble protein, *OS* periplasmic proteins by osmotic shock purification, *Su* supernatant

The gene sequence of mCsn2-SP-linked hSOD was synthesized and subsequently inserted into the pET-11a vector, which was then transformed into *E. coli* BL21(DE3) cells. An attempt was also made to induce expression at 30 °C, but no hSOD expression was detected (data not shown). Therefore, the experiment was performed at 20 °C only. The results of inducing protein expression at 20 °C for 24 h are shown in Fig. 3, where two distinct bands were observed, corresponding to pre-hSOD and mature hSOD forms, respectively. The predicted molecular weight of the pre-hSOD protein was 19.1 kDa. That of the mature hSOD protein was 15.8 kDa. To confirm that mature hSOD migrated to the periplasm, the osmotic shock method was used. The majority of mature hSOD that migrated to the periplasmic region was soluble, while the pre-hSOD that remained in the cytoplasmic region was insoluble. No protein was secreted into the supernatant.

## Discussion

In the field of biotechnology, it has become increasingly apparent that there is no universal signal peptide that can universally enhance the optimal secretion of desired target proteins across different bacterial expression hosts (Freudl, 2018). The first systematic study to explore the effects of signal peptide variations on the secretion production of heterologous proteins was reported by Brockmeier et al. (Brockmeier et al., 2006). One surprising outcome of this study was that signal peptides optimal for one protein were often ineffective for another, underscoring the necessity for a specific combination between the signal peptide and the target protein. Therefore, the discovery of new signal peptides provides fresh options for the production of recombinant proteins, highlighting the tailored approach needed in optimizing secretion systems for diverse proteins.

In this study, we investigated the genetic characterization of chitosanase in two *B. subtilis* strains and produced it as a recombinant protein in *E. coli* to determine the secretion properties of the signal peptide. We compared the genetic profile of the known 168CSN with that of CH1CSN and CH2CSN, as shown in Fig. 1A. The results show that there is a small difference of only 1 bp between 168CSN and CH1CSN, while there is a larger difference of 30 bp in CH2CSN. There is also a difference of 31 bp between CH1CSN and CH2CSN. Looking at the signal sequence alone, there is no difference between Csn168-SP and Csn1-SP, while a 20 bp difference was observed in mCsn2-SP. Interestingly, the sequences of Csn168-SP and Csn1-SP contain a repeat sequence (AAAAAGCAG) that appears to be central to the 18 bp deletion mutation identified in mCsn2-SP. This finding is consistent with the work of Bzymek and Lovett, who showed that genetic diversity in bacteria often results from genetic rearrangements in which simple sequence repeats result in a variety of insertions or deletions (Bzymek & Lovett, 2001). As shown in Fig. 1b, a comparison of the amino acid sequences of the chitosanases revealed that the mature chitosanase sequences were all identical. However, mCsn2-SP showed a deletion of six consecutive amino acids (ADFWKK) compared to Csn168-SP and Csn1-SP. Signal peptides are typically divided into three main regions: the N-region, which carries a positive charge and assists in the translocation process; the H-region, which is hydrophobic and binds to the membrane in an alpha-helix structure; and the C-region, which is polar and contains the cleavage site that releases the mature protein (Rusch & Kendall, 2007). Specially, the N-region plays a crucial role in facilitating protein translocation by interacting with the negatively charged phosphate groups in the lipid bilayer, which is vital for the effective movement of proteins. Despite the H-region and C-region of mCsn2-SP being identical to those of Csn168-SP and Csn1-SP, the N-region of mCsn2-SP exhibited a net charge decrease from + 2 to + 1 due to the deletion of six amino acids. Although the net positive charge in the N-region of a signal peptide is crucial for the efficient translocation of target proteins, an increase in net positive charge does not always result in beneficial outcomes. In fact, higher positive charges can lead to negative consequences such as protein aggregation, jamming of the secretion translocase, and activation of stress-related genes (Owji et al., 2018).

We determined the chitosanase secretion efficiency of Csn1-SP and mCsn2-SP as a function of concentration and time of IPTG treatment, a protein expression inducer in *E. coli*. SDS-PAGE showed that both forms of pre-chitosanase, in which the signal peptide is not cleaved and is located in the cytoplasm, and mature chitosanase, in which the signal peptide has been cleaved and translocated to the periplasmic region, were observed in the total protein of the cells, and some of the protein was released into the culture supernatant (Fig. 2A). The difference in length of the signal peptides resulted in the size of pre-CH1CSN (31.5 kDa) being larger in molecular weight than pre-CH2CSN (30.7 kDa), while the mature forms of CH1CSN and CH2CSN were the same size (27.4 kDa). The results show that the signal peptide of the naturally mutated CH2CSN significantly improves the efficiency of protein secretion compared to CH1CSN under all conditions used in the experiment (Fig. 2B, Table 1). This result suggests that minor alterations in the signal peptide sequence can have substantial impacts on the protein export process in bacterial systems. Consequently, these results may imply that such mutations contribute to the enhancement of genomic diversity and highlight the potential of adaptive evolutionary processes in microbial genetics (Vale et al., 2022). To determine the potential for the use of this mCsn2-SP in the production of other heterologous recombinant proteins, we ligated human-derived hSOD. Previous studies have employed the OmpA signal peptide to facilitate the translocation of hSOD to the periplasmic region in *E. coli* (Mao et al., 2010; Takahara et al., 1988). Similarly, our results demonstrated that mCsn2-SP was also effective in translocating hSOD to the periplasmic region of *E. coli*, thereby confirming its usefulness (Fig. 3).

Overall, the secretory capability of *E. coli* provides valuable advantages in terms of increased yield, simplified purification, reduced cell toxicity, improved post-translational modifications, and scalability, making it a popular choice for recombinant protein expression and production (Baldi et al., 2007). The Sec pathway is a well-known secretory pathway in *E. coli* and is involved in the secretion of various proteins. Representative proteins secreted via the Sec pathway in *E. coli* include MalE (Beena et al., 2004), OmpA (Pechsrichuang et al., 2016), OmpC (Wang et al., 2016), OmpF (Forster et al., 2013), PelB (Shi et al., 2021), and PhoA (Miksch et al., 2008) signal peptides. As signal peptides in the Sec pathway, Csn1-SP and mCsn2-SP were also identified. We also identified a mutant chitosanase signal peptide, mCsn2-SP, and successfully demonstrated secretion of recombinant proteins in *E. coli*. This study confirmed whether mCsn2-SP has a higher secretion efficiency than Csn1-SP in *E. coli.* A previous study by another group showed that the *E. coli* OmpA signal sequence was more effective in both expression and secretion than the native *Bacillus* signal peptide (Pechsrichuang et al., 2016). In Gram-negative bacteria, such as *E. coli*, the Sec pathway plays a key role in protein secretion. It is also known to recognize cleavage sites and selectively target the Sec translocase. Nevertheless, these results confirm that the signal peptide, originally from Gram-positive bacteria, operates effectively even when interacting with distinct receptors within the secretion system of Gram-negative bacteria.

Collectively, we compared the nucleotide and amino acid sequences of CH1CSN and CH2CSN from *B. subtilis* and found that a repetitive sequence in the Csn1-SP resulted in an 18 bp base deletion in the mCsn2-SP. This was manifested as a deletion of 6 aa at the end of the N-region of mCsn2-SP. When CH1CSN and CH2CSN were produced as recombinant proteins in *E. coli*, the secretion rate of CH2CSN was found to be superior to that of CH1CSN under all expression induction conditions, demonstrating that the deletion of the N-region that affects translocation within mCsn2-SP is an evolutionary consequence of its evolution relative to Csn1-SP. Also, we have confirmed that mCsn2-SP can also be applied to the production of heterologous proteins.

## Supplementary Information

Below is the link to the electronic supplementary material.

Supplementary file1 (PDF 139 KB)
