# Supplementary figures and images for: Comparative Secretory Efficiency of Two Chitosanase Signal Peptides from Bacillus subtilis in Escherichia coli
Source: J Microbiol. 2024 Nov 25;62(12):1155–64. doi: 10.1007/s12275-024-00186-1 (PMC11652591; doi:10.1007/s12275-024-00186-1)

**Fig. S1** Signal peptide diagnostics of CH1CSN and CH2CSN using SignalP 6.0 tool

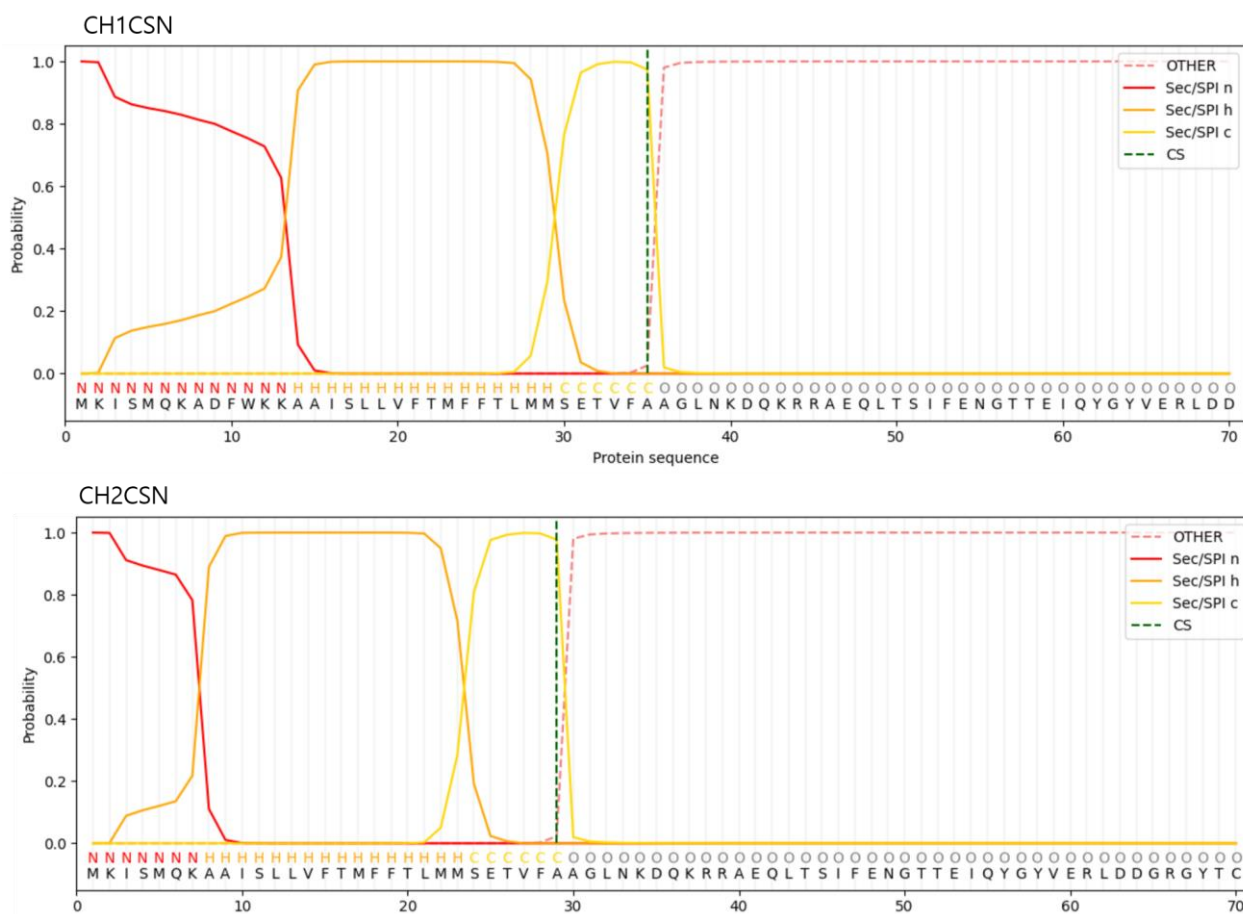

Supplement: Supplementary file 1 — Supplementary file1 (PDF 139 KB) [file 12275_2024_186_MOESM1_ESM.pdf]
